# Supplementary material for: Outpatient antibiotic prescription rate and pattern in the private sector in India: Evidence from medical audit data
Source: PLoS One. 2019 Nov 13;14(11):e0224848. doi: 10.1371/journal.pone.0224848 (PMC6853304; doi:10.1371/journal.pone.0224848)
Supplement: S1 Table — (DOCX) [file pone.0224848.s001.docx]

**S1 Table. Prescription rate per 1000 per year for systemic antibiotic, by age group in India, 2013-2014**

| Age groups | Tetracycline (J01A) | Amphenicols (J01B) | Beta-Lactams, Penicillin (J01C) | Beta-Lactams, Cephalosporins (J01D) | Sulfonamides & Trimethoprim (J01E) | Macrolides & Lincosamides (J01F) | Aminoglycosides (J01G) | Quinolones (J01M) | Antibacterial, Combinations (J01R) | Other Antibacterial (J01X) | Unclassifiable | Total |
| --- | --- | --- | --- | --- | --- | --- | --- | --- | --- | --- | --- | --- |
| 0-4 (years) | 0 | 1 | 208 | 283 | 7 | 94 | 13 | 25 | 1 | 3 | 0 | 636 |
| 5-9 (years) | 1 | 1 | 109 | 145 | 3 | 51 | 4 | 23 | 1 | 1 | 0 | 340 |
| 10-19 (years) | 8 | 1 | 63 | 109 | 2 | 47 | 5 | 41 | 3 | 1 | 1 | 280 |
| 20-29 (years) | 19 | 2 | 87 | 157 | 2 | 64 | 10 | 88 | 7 | 4 | 2 | 442 |
| 30-39 (years) | 20 | 2 | 90 | 164 | 2 | 63 | 11 | 95 | 8 | 5 | 3 | 464 |
| 40-49 (years) | 14 | 2 | 87 | 154 | 2 | 54 | 11 | 89 | 7 | 6 | 2 | 429 |
| 50-59 (years) | 12 | 2 | 89 | 166 | 2 | 53 | 11 | 92 | 7 | 8 | 2 | 445 |
| 60-64 (years) | 10 | 1 | 59 | 116 | 2 | 35 | 9 | 63 | 5 | 6 | 1 | 307 |
| 65+(years) | 11 | 1 | 76 | 160 | 2 | 44 | 11 | 91 | 6 | 11 | 2 | 415 |
| Total (%) | 12 (2.9) | 2 (0.5) | 94 (22.8) | 158 (38.3) | 3 (0.7) | 58 (14.1) | 9 (2.2) | 67 (16.3) | 5 (1.2) | 4 (1.0) | 2 (0.5) | 412 (100) |
