# Supplementary material for: Outpatient antibiotic prescription rate and pattern in the private sector in India: Evidence from medical audit data
Source: PLoS One. 2019 Nov 13;14(11):e0224848. doi: 10.1371/journal.pone.0224848 (PMC6853304; doi:10.1371/journal.pone.0224848)
Supplement: S2 Table — (DOCX) [file pone.0224848.s002.docx]

**S2 Table. Top 10 diagnoses for antibiotic prescriptions, by age group, India, 2013-2014**

| Rank | 0 yrs- 4 yrs (%) | 5yrs-9yrs (%) | 10yrs-19yrs (%) | 20yrs-29yrs (%) | 30yrs-39yrs (%) | 40yrs-49yrs (%) | 50yrs-59yrs (%) | 60yrs-64yrs (%) | 65+ yrs (%) |
| --- | --- | --- | --- | --- | --- | --- | --- | --- | --- |
| 1 | Acute upper respiratory infections (25.48) | Acute upper respiratory infections (24.23) | Acute upper respiratory infections (24.06) | Acute upper respiratory infections (19.45) | Acute upper respiratory infections (17.72) | Acute upper respiratory infections (16.56) | Acute upper respiratory infections (15.64) | Acute upper respiratory infections (13.35) | Acute upper respiratory infections (12.19) |
| 2 | Unspecified acute lower respiratory infection (24.72) | Unspecified acute lower respiratory infection (17.77) | Unspecified acute lower respiratory infection (12.94) | Unspecified acute lower respiratory infection (9.03) | Unspecified acute lower respiratory infection (9.67) | Unspecified acute lower respiratory infection (9.59) | Unspecified acute lower respiratory infection (9.90) | Unspecified acute lower respiratory infection (9.72) | Unspecified acute lower respiratory infection (10.13) |
| 3 | Acute nasopharyngitis (6.13) | Cough (5.26) | Acute pharyngitis (4.97) | Other disorders of urinary system (7.73) | Other disorders of urinary system (8.06) | Other disorders of urinary system  (7.68) | Other disorders of urinary system (7.59) | Other disorders of urinary system (7.32) | Other disorders of urinary system (9.00) |
| 4 | Cough (6.13) | Acute nasopharyngitis (5.01) | Acute nasopharyngitis (4.73) | Acute nasopharyngitis (5.00) | Acute pharyngitis (4.67) | Cough (4.57) | Cough (3.93) | Asthma (4.39) | Asthma (4.36) |
| 5 | Acute bronchitis (5.05) | Acute bronchitis (4.03) | Cough (4.64) | Acute pharyngitis (4.79) | Cough (4.54) | Acute nasopharyngitis (4.37) | Acute bronchitis (3.71) | Cough (3.94) | Acute bronchitis (4.18) |
| 6 | Acute pharyngitis (2.20) | Acute pharyngitis (3.87) | Other disorders of urinary system (3.81) | Cough (4.62) | Acute nasopharyngitis (4.51) | Acute pharyngitis (4.01) | Asthma (3.56) | Acute bronchitis (3.82) | Cough (2.88) |
| 7 | Asthma (1.85) | Acute tonsillitis (3.27) | Injury, poisoning and certain other consequences of external causes (3.20) | Injury, poisoning and certain other consequences of external causes (3.14) | Acute bronchitis (2.90) | Acute bronchitis (3.39) | Acute pharyngitis (3.16) | Acute nasopharyngitis (2.75) | Other chronic obstructive pulmonary disease (2.78) |
| 8 | Other disorders of urinary system (1.68) | Other disorders of urinary system (2.45) | Acute bronchitis (2.81) | Cutaneous abscess, furuncle and carbuncle (2.65) | Injury, poisoning and certain other consequences of external causes (2.79) | Asthma (2.58) | Acute nasopharyngitis (3.12) | Acute pharyngitis (2.23) | Acute nasopharyngitis (2.00) |
| 9 | Cutaneous abscess, furuncle and carbuncle  (1.47) | Injury, poisoning and certain other consequences of external causes (2.36) | Acute tonsillitis (2.76) | Acute bronchitis (2.41) | Cutaneous abscess, furuncle and carbuncle (2.46) | Cutaneous abscess, furuncle and carbuncle (2.54) | Cutaneous abscess, furuncle and carbuncle (2.38) | Cutaneous abscess, furuncle and carbuncle (1.92) | Hyperplasia of prostate (1.93) |
| 10 | Chronic rhinitis, nasopharyngitis and pharyngitis (1.39) | Cutaneous abscess, furuncle and carbuncle (2.08) | Cutaneous abscess, furuncle and carbuncle  (2.59) | Other female pelvic inflammatory diseases (1.69) | Asthma (1.99) | Injury, poisoning and certain other consequences of external causes (2.51) | Injury, poisoning and certain other consequences of external causes (2.23) | Injury, poisoning and certain other consequences of external causes (1.86) | Acute pharyngitis (1.90) |
