# Supplementary material for: Outpatient antibiotic prescription rate and pattern in the private sector in India: Evidence from medical audit data
Source: PLoS One. 2019 Nov 13;14(11):e0224848. doi: 10.1371/journal.pone.0224848 (PMC6853304; doi:10.1371/journal.pone.0224848)
Supplement: S3 Table — (DOCX) [file pone.0224848.s003.docx]

**S3 Table. Distribution of systemic antibiotic prescriptions by disease conditions and antibiotic classes in India, 2013-2014**

| ICD 10 | Tetracycline (J01A) | Amphenicols (J01B) | Beta-Lactams, Penicillin (J01C) | Beta-Lactams, Cephalosporins (J01D) | Sulfonamides & Trimethoprim (J01E) | Macrolides & Lincosamides (J01F) | Aminoglycosides (J01G) | Quinolones (J01M) | Antibacterial, Combinations (J01R) | Other Antibacterial (J01X) | Unclassifiable | Total (J01) | % |
| --- | --- | --- | --- | --- | --- | --- | --- | --- | --- | --- | --- | --- | --- |
| Acute upper respiratory infections | 10,76,293 | 2,19,294 | 2,71,48,648 | 4,42,27,428 | 4,53,731 | 2,09,83,919 | 10,35,105 | 94,31,357 | 12,57,128 | 1,67,753 | 52,844 | 10,60,00,656 | 20.5 |
| Unspecified acute lower respiratory infection | 5,56,300 | 4,58,547 | 1,94,92,679 | 2,75,05,910 | 2,62,929 | 98,11,406 | 11,19,210 | 65,57,836 | 6,19,244 | 1,99,667 | 47,525 | 6,65,83,728 | 12.9 |
| Other disorders of urinary system | 2,71,009 | 45,583 | 10,36,324 | 1,06,82,925 | 2,21,637 | 4,47,740 | 15,47,055 | 1,43,92,581 | 1,67,084 | 16,84,760 | 6,47,963 | 3,04,96,698 | 5.9 |
| Cough | 8,55,233 | 2,19,579 | 76,50,335 | 81,59,224 | 86,898 | 28,55,048 | 5,82,252 | 37,11,784 | 2,47,342 | 16,903 | 17,236 | 2,43,84,598 | 4.7 |
| Acute nasopharyngitis | 7,34,648 | 1,13,589 | 65,12,537 | 78,63,553 | 1,95,964 | 27,89,751 | 6,43,416 | 47,89,198 | 1,56,567 | 8,367 | 5,526 | 2,38,07,590 | 4.6 |
| Acute pharyngitis | 2,81,981 | 33,418 | 42,81,538 | 76,00,631 | 71,300 | 57,35,920 | 1,86,017 | 16,80,660 | 2,39,544 | 13,757 | 22,581 | 2,01,24,766 | 3.9 |
| Acute bronchitis | 2,37,030 | 18,631 | 54,00,670 | 70,29,324 | 62,731 | 22,34,711 | 3,19,156 | 22,02,439 | 1,65,479 | 36,629 | 17,749 | 1,77,06,800 | 3.4 |
| Injury, poisoning and others | 1,66,851 | 19,998 | 39,43,760 | 49,93,311 | 1,38,025 | 6,15,389 | 4,13,296 | 23,54,455 | 69,040 | 1,01,620 | 35,387 | 1,28,15,745 | 2.5 |
| Cutaneous abscess and furuncle | 2,62,933 | 24,332 | 40,07,468 | 47,18,742 | 1,46,543 | 11,07,489 | 2,69,351 | 10,23,165 | 92,407 | 1,10,665 | 73,834 | 1,17,63,095 | 2.3 |
| Asthma | 2,03,276 | 23,191 | 30,77,293 | 39,96,904 | 43,350 | 20,45,870 | 2,53,159 | 17,04,996 | 1,14,432 | 48,757 | 10,755 | 1,15,11,228 | 2.2 |
| Acute tonsillitis | 55,998 | 11,899 | 25,84,224 | 27,27,054 | 32,023 | 20,83,812 | 96,138 | 4,49,823 | 66,094 | 13,736 | 23,248 | 81,20,801 | 1.6 |
| Chronic sinusitis | 95,746 | 5,312 | 13,66,027 | 23,87,845 | 20,026 | 8,52,232 | 39,889 | 10,52,549 | 76,572 | 10,946 | 7,641 | 59,07,144 | 1.1 |
| Chronic rhinitis and nasopharyngitis | 72,177 | 5,196 | 12,98,356 | 22,76,883 | 25,511 | 9,97,447 | 51,841 | 7,43,309 | 46,352 | 6,988 | 7,269 | 55,24,060 | 1.1 |
| Acute bronchiolitis | 38,212 | 1,953 | 13,53,924 | 22,04,804 | 16,377 | 5,75,951 | 67,310 | 8,93,702 | 23,682 | 7,953 | 1,925 | 51,83,868 | 1.0 |
| Abdominal and pelvic pain | 2,05,296 | 16,991 | 4,09,925 | 16,82,351 | 32,765 | 2,13,038 | 3,14,584 | 21,59,861 | 33,506 | 1,13,918 | 13,160 | 51,82,235 | 1.0 |
| Other female pelvic inflammatory diseases | 9,97,579 | 1,423 | 2,43,334 | 10,43,994 | 5,532 | 5,35,201 | 90,538 | 6,75,722 | 3,01,574 | 52,640 | 11,663 | 39,47,537 | 0.8 |
| Gastritis and duodenitis | 89,825 | 28,738 | 3,65,910 | 13,77,504 | 29,790 | 2,41,495 | 2,25,595 | 13,00,042 | 31,238 | 48,675 | 8,302 | 37,38,812 | 0.7 |
| Acne | 13,27,818 | 1,160 | 99,297 | 1,82,887 | 24,191 | 18,73,749 | 4,102 | 63,978 | 8,209 | 1,545 | 12,197 | 35,86,936 | 0.7 |
| Suppurative and unspecified otitis media | 47,553 | 4,279 | 6,20,760 | 18,02,886 | 33,932 | 3,76,006 | 74,446 | 4,03,481 | 39,778 | 12,585 | 9,064 | 34,15,706 | 0.7 |
| Other soft tissue disorders, not elsewhere classified | 1,21,410 | 49,031 | 6,13,472 | 10,51,107 | 20,813 | 1,86,028 | 1,89,449 | 11,46,569 | 9,412 | 8,599 | 9,425 | 33,95,890 | 0.7 |
| Headache | 4,11,778 | 23,707 | 3,89,921 | 10,00,864 | 14,854 | 3,03,955 | 68,258 | 9,50,231 | 18,521 | 5,457 | 3,998 | 31,87,546 | 0.6 |
| Stomatitis and related lesions | 3,20,404 | 11,832 | 7,53,931 | 10,19,434 | 47,094 | 5,24,623 | 49,891 | 3,78,808 | 24,493 | 3,436 | 7,097 | 31,33,946 | 0.6 |
| Other chronic obstructive pulmonary disease | 45,204 | 7,266 | 7,81,461 | 10,66,266 | 8,057 | 4,50,226 | 52,378 | 5,57,349 | 38,245 | 17,275 | 11,783 | 30,23,727 | 0.6 |
| Vasomotor and allergic rhinitis | 47,723 | 1,978 | 4,69,043 | 10,31,953 | 8,818 | 6,15,655 | 19,861 | 7,71,536 | 38,256 | 3,737 | 3,867 | 30,08,560 | 0.6 |
| Sickle-cell disorders | 70,568 | 3,025 | 6,85,670 | 12,12,004 | 12,128 | 6,66,629 | 30,705 | 2,60,861 | 22,399 | 8,385 | 5,932 | 29,72,374 | 0.6 |
| Gastric ulcer | 1,22,646 | 16,210 | 3,69,857 | 10,28,608 | 13,318 | 2,55,403 | 58,271 | 8,75,610 | 34,397 | 34,653 | 9,685 | 28,08,973 | 0.5 |
| Dental caries | 2,01,858 | 1,135 | 8,09,258 | 11,17,029 | 6,941 | 2,56,531 | 20,834 | 2,66,841 | 87,576 | 4,034 | 2,224 | 27,72,037 | 0.5 |
| Gingivitis and periodontal diseases | 2,76,422 | 652 | 8,19,450 | 10,85,427 | 10,840 | 2,20,923 | 29,231 | 2,54,315 | 68,843 | 5,878 | 2,580 | 27,71,981 | 0.5 |
| Other gastroenteritis and colitis of infectious and unspecified origin | 1,15,387 | 36,059 | 3,22,949 | 11,35,845 | 82,843 | 1,99,703 | 1,67,277 | 5,57,828 | 27,471 | 99,272 | 20,088 | 27,44,634 | 0.5 |
| Diseases of pulp and periapical tissues | 1,55,092 | 390 | 8,43,489 | 10,32,615 | 11,160 | 2,13,644 | 26,636 | 2,16,947 | 78,847 | 8,241 | 3,153 | 25,87,061 | 0.5 |
| Cellulitis | 32,938 | 1,140 | 8,33,247 | 10,58,355 | 4,060 | 1,39,674 | 57,463 | 2,20,382 | 46,847 | 78,257 | 2,826 | 24,72,363 | 0.5 |
| Fever of other and unknown origin | 72,820 | 54,452 | 4,33,384 | 8,90,114 | 55,431 | 2,70,700 | 72,756 | 3,49,217 | 93,825 | 69,921 | 35,122 | 23,62,620 | 0.5 |
| Others | 49,18,046 | 4,10,019 | 1,85,89,521 | 3,98,52,958 | 10,50,333 | 1,12,14,282 | 32,79,463 | 2,03,62,606 | 21,48,505 | 19,64,114 | 8,62,919 | 10,37,89,847 | 20 |
