# Supplementary material for: Outpatient antibiotic prescription rate and pattern in the private sector in India: Evidence from medical audit data
Source: PLoS One. 2019 Nov 13;14(11):e0224848. doi: 10.1371/journal.pone.0224848 (PMC6853304; doi:10.1371/journal.pone.0224848)
Supplement: Attachment — Submitted filename: Plos review v1.pdf [file pone.0224848.s005.pdf]

PLOS ONE

Outpatient antibiotic prescription rate and pattern in the private sector in India: Evidence from medical audit data

--Manuscript Draft--

Manuscript Number:	PONE-D-19-14711
Article Type:	Research Article
Full Title:	Outpatient antibiotic prescription rate and pattern in the private sector in India: Evidence from medical audit data
Short Title:	Outpatient antibiotic prescription rate and pattern in the private sector in India
Corresponding Author:	Habib Hasan Farooqui, MD<br>Public Health Foundation of India<br>Gurgaon, INDIA
Keywords:	India; antibiotic; prescription; private sector
Abstract:	The key objective of this research was to generate new evidence on outpatient antibiotic prescription rate and patterns in the private sector in India. We used 12-month period (May 2013 to April 2014) medical audit dataset from IMS Health (now IQVIA). We coded the diagnosis provided in the medical audit data to International Statistical Classification of Diseases and Related Health Problems (ICD-10) and the prescribed antibiotics for the diagnosis to Anatomic Therapeutic Chemical (ATC) classification of World Health Organization (ATC index-2016). We calculated and reported antibiotic prescription rate per 1,000 persons per year, by age groups, antibiotic class and disease conditions. Our main findings are - approximately 519 million antibiotic prescriptions were dispensed in the private sector, which translates into 412 prescriptions per 1,000 persons per year. Majority of the antibiotic prescriptions were dispensed for acute upper respiratory infections (J06) (20.4%); unspecified acute lower respiratory infection (J22) (12.8%); disorders of urinary system (N39) (6.0%); cough (R05) (4.7%); and acute nasopharyngitis (J00) (4.6%) and highest antibiotic prescription rates were observed in the age group 0-4 years. To conclude our study reports first ever country level estimates of antibiotic prescription by antibiotic classes, age groups, and ICD-10 mapped disease conditions. We observed an inappropriate and high antibiotic prescription rates for upper respiratory infections in children age less than 5 years.
Order of Authors:	Habib Hasan Farooqui, MD
	Aashna Mehta
	Sakthivel Selvaraj
Additional Information:	
Question	Response
**Financial Disclosure**<br><br>Enter a financial disclosure statement that describes the sources of funding for the work included in this submission. Review the submission guidelines for detailed requirements. View published research articles from *PLOS ONE* for specific examples.<br><br>This statement is required for submission and **will appear in the published article** if the submission is accepted. Please make sure it is accurate.	Habib Hasan Farooqui is supported by Department of Science and Technology's Public Health Research Grant to the Public Health Foundation of India. The funders had no role in study design, data collection and analysis, decision to publish, or preparation of the manuscript.

**Unfunded studies**<br>Enter: *The author(s) received no specific funding for this work.*<br><br>**Funded studies**<br>Enter a statement with the following details:<br>• Initials of the authors who received each award<br>• Grant numbers awarded to each author<br>• The full name of each funder<br>• URL of each funder website<br>• Did the sponsors or funders play any role in the study design, data collection and analysis, decision to publish, or preparation of the manuscript?<br>• **NO** - Include this sentence at the end of your statement: *The funders had no role in study design, data collection and analysis, decision to publish, or preparation of the manuscript.*<br>• **YES** - Specify the role(s) played.<br><br>* typeset	
**Competing Interests**<br><br>Use the instructions below to enter a competing interest statement for this submission. On behalf of all authors, disclose any competing interests that could be perceived to bias this work—acknowledging all financial support and any other relevant financial or non-financial competing interests.<br><br>This statement **will appear in the published article** if the submission is accepted. Please make sure it is accurate. View published research articles from *PLOS ONE* for specific examples.	The authors have declared that no competing interests exist.

**NO authors have competing interests**<br><br>Enter: *The authors have declared that no competing interests exist.*<br><br>**Authors with competing interests**<br><br>Enter competing interest details beginning with this statement:<br><br>*I have read the journal's policy and the authors of this manuscript have the following competing interests: [insert competing interests here]*<br><br>* typeset	
**Ethics Statement**<br><br>Enter an ethics statement for this submission. This statement is required if the study involved:<br><br>• Human participants<br>• Human specimens or tissue<br>• Vertebrate animals or cephalopods<br>• Vertebrate embryos or tissues<br>• Field research<br><br>Write "N/A" if the submission does not require an ethics statement.<br><br>General guidance is provided below. Consult the submission guidelines for detailed instructions. **Make sure that all information entered here is included in the Methods section of the manuscript.**	N/A

**Format for specific study types**<br><br>**Human Subject Research (involving human participants and/or tissue)**<br>• Give the name of the institutional review board or ethics committee that approved the study<br>• Include the approval number and/or a statement indicating approval of this research<br>• Indicate the form of consent obtained (written/oral) or the reason that consent was not obtained (e.g. the data were analyzed anonymously)<br><br>**Animal Research (involving vertebrate animals, embryos or tissues)**<br>• Provide the name of the Institutional Animal Care and Use Committee (IACUC) or other relevant ethics board that reviewed the study protocol, and indicate whether they approved this research or granted a formal waiver of ethical approval<br>• Include an approval number if one was obtained<br>• If the study involved *non-human primates*, add *additional details* about animal welfare and steps taken to ameliorate suffering<br>• If anesthesia, euthanasia, or any kind of animal sacrifice is part of the study, include briefly which substances and/or methods were applied<br><br>**Field Research**<br><br>Include the following details if this study involves the collection of plant, animal, or other materials from a natural setting:<br>• Field permit number<br>• Name of the institution or relevant body that granted permission	
**Data Availability**<br><br>Authors are required to make all data underlying the findings described fully available, without restriction, and from the time of publication. PLOS allows rare exceptions to address legal and ethical concerns. See the PLOS Data Policy and FAQ for detailed information.	No - some restrictions will apply

A Data Availability Statement describing where the data can be found is required at submission. Your answers to this question constitute the Data Availability Statement and **will be published in the article**, if accepted.<br><br>**Important:** Stating 'data available on request from the author' is not sufficient. If your data are only available upon request, select 'No' for the first question and explain your exceptional situation in the text box.<br><br>Do the authors confirm that all data underlying the findings described in their manuscript are fully available without restriction?	
**Describe where the data may be found in full sentences. If you are copying our sample text, replace any instances of XXX with the appropriate details.**<br><br>• If the data are **held or will be held in a public repository**, include URLs, accession numbers or DOIs. If this information will only be available after acceptance, indicate this by ticking the box below. For example: *All XXX files are available from the XXX database (accession number(s) XXX, XXX.*).<br>• If the data are all contained **within the manuscript and/or Supporting Information files**, enter the following: *All relevant data are within the manuscript and its Supporting Information files.*<br>• If neither of these applies but you are able to provide **details of access elsewhere**, with or without limitations, please do so. For example:<br><br>*Data cannot be shared publicly because of [XXX]. Data are available from the XXX Institutional Data Access / Ethics Committee (contact via XXX) for researchers who meet the criteria for access to confidential data.*<br><br>*The data underlying the results presented in the study are available from (include the name of the third party*	The data underlying the results presented in the study are available from the IMS Health (now IQVIA). The IMS Healtth can be approached for data access through their website (https://www.iqvia.com/locations/india).

Powered by Editorial Manager® and ProduXion Manager® from Aries Systems Corporation

and contact information or URL).<br>• This text is appropriate if the data are owned by a third party and authors do not have permission to share the data.<br><br>* typeset	
Additional data availability information:	

Manuscript

Click here to download Manuscript PLOSOne_Manuscript File_24th May.docx

Outpatient antibiotic prescription rate and pattern in the private sector in India: Evidence from medical audit data

Habib Hasan Farooqui[1]*, Aashna Mehta[2], Sakthivel Selvaraj[2]

[1] Indian Institute of Public Health –Delhi, Public Health Foundation of India, Gurugram, Haryana, India

[2] Health Economics, Financing and Policy, Public Health Foundation of India, Gurugram, Haryana, India

* Corresponding author:

Email: drhabibhasan@gmail.com (HHF)

Abstract

The key objective of this research was to generate new evidence on outpatient antibiotic prescription rate and patterns in the private sector in India. We used 12-month period (May 2013 to April 2014) medical audit dataset from IMS Health (now IQVIA). We coded the diagnosis provided in the medical audit data to International Statistical Classification of Diseases and Related Health Problems (ICD-10) and the prescribed antibiotics for the diagnosis to Anatomic Therapeutic Chemical (ATC) classification of World Health Organization (ATC index-2016). We calculated and reported antibiotic prescription rate per 1,000 persons per year, by age groups, antibiotic class and disease conditions. Our main findings are - approximately 519 million antibiotic prescriptions were dispensed in the private sector, which translates into 412 prescriptions per 1,000 persons per year. Majority of the antibiotic prescriptions were dispensed for acute upper respiratory infections (J06) (20.4%); unspecified acute lower respiratory infection (J22) (12.8%); disorders of urinary system (N39) (6.0%); cough (R05) (4.7%); and acute nasopharyngitis (J00) (4.6%) and highest antibiotic prescription rates were observed in the age group 0-4 years. To conclude our study reports first ever country level estimates of antibiotic prescription by antibiotic classes, age groups, and ICD-10 mapped disease conditions. We observed an inappropriate and high antibiotic prescription rates for upper respiratory infections in children age less than 5 years.

Introduction

India is considered to be one of the top users of antibiotics. Previous research had reported that per capita antibiotic consumption in the retail sector in India has increased by around 22%, from 13.1 DID in 2008 to 16.0 DID, in span of five years (2012 to 2016).[1] Current evidence suggests that between 2000 and 2015, antibiotic consumption increased from 3.2 to 6.5 billion DDDs (103%) while the antibiotic consumption rate increased from 8.2 to 13.6 DDDs per 1,000 inhabitants per day (63%) in India.[2]

Literature suggests that high burden of infectious disease is one of the reasons for high antibiotic use in India. Earlier Million Death Study (MDS) had reported that diseases of infectious origin such as pneumonia and diarrhea accounted for around 50% (0·67 million of 1·34 million) of all deaths in children aged less than 5 years in India.[3] However, inappropriate use of antibiotics cannot be ruled out. In spite of the fact that majority of upper respiratory infections like common cold and nasopharyngitis are viral in origin, self limiting in nature and do not require antibiotic therapy,[4-6] literature on antibiotic use and prescription pattern from India indicate high rate of antibiotic prescriptions for respiratory infections in the primary care.[7-9] Previous research had also reported relatively high antibiotic prescription rate in the private health facilities as compared to public health facilities.[8 10] These studies also highlighted frequent use of expensive newer classes of antibiotics as compared to the older ones in the private sector.[8-11] One of the reasons for such a trend is dominance of private sector in funding and provisioning of the health care in India. As per the National Sample Survey

(NSS) nearly 75% of all outpatient visits and about 62% of hospitalization episodes occurred in private health delivery system in the year 2014.[12] Furthermore, households largely buy medicines directly from retail pharmacies as prescribed by the general practitioners in the private sector.[13] Evidence suggests that, of total INR 1338[14] per capita spending on medicines in India, around INR 1208 per capita was spent by households as out of pocket expenditure, which indicates that government's contribution is barely 10 percent.

The health system elements outlined above clearly demonstrate the role and relevance of general practitioners in the private sector in rational medicine use and in particular antibiotic use. Previous research involving micro level surveys revealed several facets of inappropriate medicines use in the Indian context.[8 9 15-17] However, none of them were truly representative of general practitioners because of their limited sample sizes and geographical locations. We conducted this research to generate new evidence on outpatient antibiotic (J01) prescription rate and patterns in the private sector in India. We also performed additional analysis to report age-specific and disease-specific differences by different antibiotic classes.

Material and Methods

Data source and setting

We examined prescription rate and patterns of antibiotics (J01) of general practitioners working in the private sector in India with the help of

Intercontinental Medical Services medical audit data (now IQVIA) for 12 month period (May 2013 to April 2014).[18] IMS Health (now IQVIA) is a for-profit organisation that collects and provides data and information on pharmaceutical market intelligence in over 100 countries around the world. The medical audit data tracks prescription by private practitioners practicing allopathic system of medicine. This data is collected from a panel comprising of 4600 doctors, which include general practitioners (MBBS, Bachelor of Medicine, Bachelor of Surgery), non-MBBS general practitioners, and other medical specialties (such as dentists, pediatrics, gynecology, dermatology, and others) from 23 metropolitan areas (population more than 1 million), 128 class 1 towns (population more than 100,000) and 1A towns (population less than 100,000) of India. The data is then extrapolated to reflect the private sector prescription pattern in the entire country.

This database provides information on patient characteristics such as age, gender, symptoms, diagnosis and medicines prescribed. The data organizes medicines according to anatomical therapeutic classification (ATC) of the European Pharmaceutical Market Research Association (EphMRA) but not to the World Health Organisation's ATC classification. Also, the diagnosis are not coded to International Statistical Classification of Diseases and Related Health Problems (ICD-10). Furthermore, the data does not capture the public sector prescriptions and therefore our analysis only reflects outpatient antibiotic prescription patterns in the private sector in the country.

Outcome measure

Our primary outcome measure was antibiotic prescription rate per 1,000 persons per year. We also estimated and reported age-specific and disease-specific antibiotic prescription by antibiotic classes.

Statistical analysis

We coded the diagnosis provided in the medical audit data to disease classifications based on the International Statistical Classification of Diseases and Related Health Problems, 10th Revision (ICD-10 classification; version: 2016)[19] and antibiotics prescribed for the related diagnosis to 3rd level of Anatomic Therapeutic Chemical (ATC) classification as per the methodology proposed by World Health Organization's Collaborating Centre (WHOCC) of Drug Statistics Methodology's (ATC index-2016).[20]

The utilization of antibiotics (ATC code: J01) was measured in terms of the annual prescription rate, i.e. number of antibiotic prescriptions divided by the 1000 person years. The population estimates were obtained from report of the technical group on population projections constituted by the National Commission on Population.[21] The age-group classification was determined by the medical audit data availability. The prescriptions were divided into the following antibiotic subgroups (ATC codes): tetracyclines (J01A), amphenicols (J01B), penicillins (J01C), other beta-lactams, cephalosporins (J01D), sulfonamides & trimethoprim (J01E), macrolides, lincosamides & streptogramins (J01F), aminoglycosides (J01G), Quinolones (J01M), combinations of

antibacterials (J01R), other antibacterials (J01X). Unclassifiable antibiotics were pooled in the subgroup 'others'.

We used per 1000 person denominator in contrast to individuals because the data was available only for prescription per person and not for an individual for the entire year. Antibiotic prescription rates are more appropriate indicator to describe antibiotic use[22] as compared to defined daily doses (DDD) per person since antibiotic dose depends on a patient's age and body weight. We analyzed and reported antibiotic use by age groups and disease conditions (based on ICD-10 classification) expressed as annual prescription rate per 1,000 persons for each class of antibiotics. We used software STATA 14.0 to perform statistical analysis.

Results

Antibiotic prescribing pattern

We present new evidence on outpatient antibiotic prescription rates and pattern in the private sector in India. Around 519 million antibiotic prescriptions were dispensed in 2014, which translate into 412 prescriptions per 1,000 persons per year. The antibiotic prescription rates were highest for the children age 0-4 years (636 prescriptions per 1,000 persons) and lowest in the age group 10-19 years (280 prescriptions per 1,000 persons) (Fig 1). It may also be noted that across all age groups beta-lactam, cephalosporin (J01D) had the highest

prescription rates (38.3% of all antibiotic prescriptions) followed by beta-lactam, penicillin (J01C) (22.8%) and quinolone (J01M) (16.3%).

Antibiotic prescribing across clinical diagnoses

Of 519 million antibiotic prescriptions, majority were dispensed for the diseases of respiratory system (55%), followed by diseases of genitourinary system (10%), and symptoms, signs and abnormal clinical findings (9%) (Table 1).

Table 1. Distribution of outpatient antibiotic prescriptions in India, by disease conditions, 2013-2014

ICD code	ICD label	Number of prescriptions	Percentage (%)	Prescription rate per 1000 persons per year
1	Certain infectious and parasitic diseases	16,820,293	3.24	13.35
3	Diseases of the blood and blood-forming organs	1,158,125	0.22	0.92
4	Endocrine, nutritional and metabolic diseases	4,566,575	0.88	3.63
5	Mental and behavioral disorders	967,392	0.19	0.77
6	Diseases of the	1,491,169	0.29	1.18

	nervous system			
7	Diseases of the eye and adnexa	3,685,540	0.71	2.93
8	Diseases of the ear and mastoid process	8,653,317	1.67	6.87
9	Diseases of the circulatory system	8,472,213	1.63	6.73
10	Diseases of the respiratory system	286,059,212	55.09	227.10
11	Diseases of the digestive system	28,532,251	5.49	22.65
12	Diseases of the skin and subcutaneous tissue	20,917,567	4.03	16.61
13	Diseases of the musculoskeletal system	5,973,066	1.15	4.74
14	Diseases of the genitourinary system	51,791,862	9.97	41.12
15	Pregnancy, childbirth and the puerperium	977,532	0.19	0.78
17	Congenital malformations, deformations	114,892	0.02	0.09

18	Symptoms, signs and abnormal clinical and laboratory	44,506,708	8.57	35.33
19	Injury, poisoning and external causes	33,398,837	6.43	26.52
20	External causes of morbidity and mortality	37,318	0.01	0.03
21	Factors influencing health status	1,118,282	0.22	0.89
	Total	519,242,151	100.00	412.23

As per the ICD-10 classification, following top ten disease conditions contributed approximately 63% of the antibiotic prescriptions - acute upper respiratory infections (J06) (20.4%), unspecified acute lower respiratory infection (J22) (12.8%), disorders of urinary system (N39) (6.0%), cough (R05) (4.7%), acute nasopharyngitis (J00) (4.6%), acute pharyngitis (J02) (3.9%), acute bronchitis (J20) (3.4%), injury, poisoning and others (T14) (2.5%), cutaneous abscess and furuncle (L02) (2.3%), and asthma (J45) (2.2%) (Fig 2). It may be further noted that cephalosporins (J01D) were the most commonly prescribed antibiotic across all diagnoses with the exception of disorders of urinary system where quinolones (J01M) were more commonly prescribed. (For full information on antibiotic prescription rates across disease conditions and antibiotic classes see supplementary file (S1 and S2 Table).

Figure legends:

Fig1: Outpatient antibiotic prescription rate per 1000 persons per year, by age groups and antibiotic classes, India (2013-2014)

Fig 2: Number of antibiotic prescriptions, by disease conditions and antibiotic classes, India (2013-2014)

Discussion

To the best of our knowledge, this study provides first ever country level estimates of outpatient antibiotic prescription rates and patterns in the private sector. Our findings illustrate significant variations in antibiotic prescription rates across age groups, by disease conditions (ICD-10 classification) and by antibiotic classes (ATC classification). Earlier Von Bockel et al. and Klein et al. had reported estimates of antibiotic consumption in India through use of pharmaceutical sales data[2 23]. However, their study did not provided information on antibiotic use by disease conditions and age groups. While other prescription analysis based studies from India had reported antibiotic utilization in public and private sector health facilities[8-11 15 24], they were not nationally representative because of limited sample sizes and geographical locations.

Our estimates suggest high proportion of antibiotic prescription for upper respiratory tract infections (acute upper respiratory infections (J06) (20.4%), cough (R05) (4.7%), acute nasopharyngitis (J00) (4.6%), and acute pharyngitis (J02) (3.9%)). These infections are viral in origin and are generally self-limiting in nature; hence this high rate of antibiotic prescription is inappropriate in the

light of evidence based medicine and standard treatment guidelines. Previous research on prescription practices had also highlighted the problem of inappropriate use of broad-spectrum antibiotics in India. For example, Kotwani et al had reported that in the private sector, not only the antibiotic prescription rates were higher but also the choice of antibiotics for the treatment of uncomplicated respiratory infections was inappropriate.[9] Chandy et al had reported widespread use of fluoroquinolone, especially by general practitioners[10] in the private sector. Other studies have reported high cephalosporin use in urban hospitals and pharmacy shops.[10 25]

Previous research shows that that inappropriate antibiotic use is linked to supply-side incentives, which lead to over prescription of antibiotics in private sector.[15 24 26] This problem of inappropriate use of antibiotics get accentuated multifold because of limited access to care and medicines in the public health system[27] which forces patients to seek care in private sector. Limited access to medicines in public sector also results in over the counter purchase of antibiotics, which is a major driver of inappropriate use in India. Laxminarayanan et al had reported that non-prescription sales of carbapenems in India are among the highest in the world and contribute to growing carbapenem resistance.[28] Although, over the counter access of antibiotics is a complex problem in India on account of the fact that insufficient access and delays in access to antibiotics cause more deaths than antibiotic resistance,[29] numerous have reported increasing level of antimicrobial resistance for last resort antibiotics like carbapenem. Using a private laboratory network data on patient blood cultures collected over a 7-year period from January 2008 to

December, 2014, Gandra et al reported increased carbapenem resistance in *Escherichia coli* and *Klebsiella pneumonia.*[30] Other hospital based retrospective studies have also reported rise in carbapenem resistant isolates of *E. coli* from 10% to 13% during 2008 to 2013[28] and increase in carbapenem resistance among *K. pneumoniae* from 2% to 52% during 2002 to 2009[31]. Evidence also suggest that inappropriate antibiotic use not only has profound impact on antimicrobial resistance[32] but also on treatment cost because of drug resistant organism.[33]

Our analyses suggest that antibiotic prescription rates (412 prescriptions per 1000 persons in 2014) in India are significantly lower than various European nations. For example, antibiotic prescription rates in Italy (957 prescriptions per 1000 persons), Germany (561 prescriptions per 1000 persons), UK (555 prescriptions per 1000 persons), Denmark (481 prescriptions per 1000 persons)[34] and Greece (1100 antibiotics per 1000 person)[25] are much higher than India. Our findings for antibiotic prescription are consistent with antibiotic sales in India. Our previous analysis on pharmaceutical sales data had also suggested that the antibiotic consumption (16.0 DID) in India was significantly below the mean antibiotic consumption (21.5 DID) of the European countries.[1] However, antibiotic prescription rates for certain antibiotic classes are on higher side in India as compared to developed nations. For example, the percentage of prescriptions with cephalosporins and quinolones (38.2% and 16.3%) in India were significantly higher than USA (14.0% and 12.7%)[35], and also Greece (32.9% and 0.5%).[25] Such unusually high prescription rate of beta-lactams-penicillins and cephalosporins in uncomplicated upper respiratory infections in children is

in stark contrast to the prescription rates and pattern reported from European countries.[34] The potential reasons for high prescription rates of broad spectrum antibiotics like cephalosporins and quinolones are not only high burden of infectious diseases, but also lack of diagnostic support services and inadequate training of the physicians[10]. Literature also suggests that perceived demand and expectation from the patients, influence from medical representatives and inadequate knowledge influences doctors decisions to prescribe antibiotics.[36] Given the fee-for-service payment mechanisms followed by private practitioners, prescriptions and dispensing of medicines are often subject to influence by medical representatives. Previous research has highlighted that inappropriate use of medicines is rampant in less regulated health markets[37] and can take several forms: overuse, underuse, misuse, and unnecessary expensive use. This inappropriate use of antibiotics not only poses health risk on individuals and community in form of antimicrobial resistance but also leads to inefficient use of limited financial resources at household level.

To address the problems related to inappropriate use of antibiotics government of India has deployed multipronged strategy. This includes setting up treatment guidelines for antimicrobial use[38] and multi-centric surveillance for tracking antimicrobial resistance[39]. In addition, over-the-counter (OTC) sales of 3rd and 4th generation antibiotics are now regulated through Schedule H1 of Drugs & Cosmetics Rules.[40] Furthermore, to reduce burden of pneumonia and diarrhea and the demand for antibiotics, new vaccines have been introduced in the universal immunization program[41]. However, success of these strategic measures in terms of achieving intended objectives is yet to be demonstrated.

Considering that more than 519 million antibiotic prescriptions were dispensed in India during one year period (2013-2014), an antibiotic stewardship program or an intervention with a potential to reduce antibiotic prescription rates by just 10 % would result in 52 million less antibiotic prescriptions and a commensurate reduction in healthcare costs and potentially antimicrobial resistance rates. Our analysis suggest around 100 million prescriptions were dispensed for acute upper respiratory tract infections (ARTI) alone, antibiotic stewardship programs directed towards diagnosis and treatment of ARTI could result into significant reduction in antibiotic use.

Our study has certain limitations. The scope of our study was limited to analysis of antibiotic prescription pattern in the private sector, as we had no access to public sector prescription data. Therefore, the study is not representative of the prescriptions generated in public sector facilities. This may have resulted in an underestimation of antibiotic prescription rates albeit only marginally, since more than 80% of the population seeks care in private sector and approximately 90% of medicine expenditure occurs in private sector. In addition, a small proportion (<1%) of antibiotic prescriptions sometimes got mapped to completely unrelated diagnosis because IMS medical audit data is coded in such a way that antibiotics on prescriptions gets mapped to every differential diagnosis (related or completely unrelated) on the prescription.

Conclusions

This research work provided the first ever country level estimates on antibiotic prescription rate and pattern in the outpatient general practice in the private sector. Overall antibiotic prescription rates in India are still much lower than Europe. However, prescription rates for broad-spectrum beta-lactam antibiotics are much higher as compared to European nations, especially in children. Approximately one-fifth antibiotic prescriptions were dispensed for upper respiratory infections, which rarely require an antibiotic therapy. Our findings highlight that primary care physicians in the private sector can play a key role in reducing antibiotic misuse and overuse. Our research findings also provide critical information to target antimicrobial stewardship programs to specific constituencies and stakeholders. This baseline information can also be used as a benchmark for measuring the impact of current and future interventions directed towards reducing inappropriate antibiotic use.

Acknowledgments

The authors would like to thank Ms. Manushi Sharma for the data coding support.

Reference

1. Farooqui HH, Selvaraj S, Mehta A, et al. Community level antibiotic utilization in India and its comparison vis-a-vis European countries: Evidence from pharmaceutical sales data. *PLoS One* 2018;13(10):e0204805. doi: 10.1371/journal.pone.0204805

2. Klein EY, Van Boeckel TP, Martinez EM, et al. Global increase and geographic convergence in antibiotic consumption between 2000 and 2015. *Proc Natl Acad Sci U S A* 2018;115(15):E3463-E70. doi: 10.1073/pnas.1717295115

3. Million Death Study C, Bassani DG, Kumar R, et al. Causes of neonatal and child mortality in India: a nationally representative mortality survey. *Lancet* 2010;376(9755):1853-60. doi: 10.1016/S0140-6736(10)61461-4

4. Nyquist AC, Gonzales R, Steiner JF, et al. Antibiotic prescribing for children with colds, upper respiratory tract infections, and bronchitis. *JAMA* 1998;279(11):875-7.

5. Ashworth M, Latinovic R, Charlton J, et al. Why has antibiotic prescribing for respiratory illness declined in primary care? A longitudinal study using the General Practice Research Database. *J Public Health (Oxf)* 2004;26(3):268-74. doi: 10.1093/pubmed/fdh160

6. Huang N, Morlock L, Lee CH, et al. Antibiotic prescribing for children with nasopharyngitis (common colds), upper respiratory infections, and bronchitis who have health-professional parents. *Pediatrics* 2005;116(4):826-32. doi: 10.1542/peds.2004-2800

7. Pathak D, Pathak A, Marrone G, et al. Adherence to treatment guidelines for acute diarrhoea in children up to 12 years in Ujjain, India--a cross-sectional prescription analysis. *BMC Infect Dis* 2011;11:32. doi: 10.1186/1471-2334-11-32

8. Kotwani A, Chaudhury RR, Holloway K. Antibiotic-prescribing practices of primary care prescribers for acute diarrhea in New Delhi, India. *Value Health* 2012;15(1 Suppl):S116-9. doi: 10.1016/j.jval.2011.11.008

9. Kotwani A, Holloway K. Antibiotic prescribing practice for acute, uncomplicated respiratory tract infections in primary care settings in New Delhi, India. *Trop Med Int Health* 2014;19(7):761-8. doi: 10.1111/tmi.12327

10. Chandy SJ, Thomas K, Mathai E, et al. Patterns of antibiotic use in the community and challenges of antibiotic surveillance in a lower-middle-income country setting: a repeated cross-sectional study in Vellore, South India. *J Antimicrob Chemother* 2013;68(1):229-36. doi: 10.1093/jac/dks355

11. Sharma M, Damlin A, Pathak A, et al. Antibiotic Prescribing among Pediatric Inpatients with Potential Infections in Two Private Sector Hospitals in Central India. *PLoS One* 2015;10(11):e0142317. doi: 10.1371/journal.pone.0142317

12. Mackintosh M, Channon A, Karan A, et al. What is the private sector? Understanding private provision in the health systems of low-income and middle-income countries. *Lancet* 2016;388(10044):596-605. doi: 10.1016/S0140-6736(16)00342-1

13. Reddy K. High Level Expert Group (HLEG) report on Universal Health Coverage (UHC) for India. . New Delhi: Public Health Foundation of India, 2011.

14. GOI. National Health Accounts, India (2013-14). New Delhi: Ministry of Health and Family Welfare, 2016.

15. Kshirsagar MJ, Langade D, Patil S, et al. Prescribing patterns among medical practitioners in Pune, India. *Bull World Health Organ* 1998;76(3):271-5.

16. Phadke AR. The quality of prescribing in an Indian district. *Natl Med J India* 1996;9(2):60-5.

17. Shet A SS, Forsberg BC. Pharmacy-based dispensing of antimicrobial agents without prescription in India: appropriateness and cost burden in the private sector. *Antimicrobial Resistance and Infection Control* 2015;4(55)

18. IMS. IMS Heath.: IMS Health; 2016 [Available from: http://www.imshealth.com/en/about-us/our-company accessed 3/9/2016.

19. WHO. International Statistical Classification of Diseases and Related Health Problems 10th Revision 2016 [Available from: http://apps.who.int/classifications/icd10/browse/2016/en2016.

20. WHOCC. WHO Collaborating Centre for Drug Statistics Methodology 2015 [Available from: https://www.whocc.no/atc_ddd_index/2016.

21. GOI. Population Projection for India and States 2001-2026. New Delhi: OFFICE OF THE REGISTRAR GENERAL & CENSUS COMMISSIONER., 2006.

22. Clavenna A, Bonati M. Differences in antibiotic prescribing in paediatric outpatients. *Arch Dis Child* 2011;96(6):590-5. doi: 10.1136/adc.2010.183541

23. Van Boeckel TP, Gandra S, Ashok A, et al. Global antibiotic consumption 2000 to 2010: an analysis of national pharmaceutical sales data. *Lancet Infect Dis* 2014;14(8):742-50. doi: 10.1016/S1473-3099(14)70780-7

24. Gopalakrishnan S, Ganeshkumar P, Katta A. Assessment of prescribing practices among urban and rural general practitioners in Tamil Nadu. *Indian J Pharmacol* 2013;45(3):252-7. doi: 10.4103/0253-7613.111931

25. Kourlaba G, Kourkouni E, Spyridis N, et al. Antibiotic prescribing and expenditures in outpatient paediatrics in Greece, 2010-13. *J Antimicrob Chemother* 2015;70(8):2405-8. doi: 10.1093/jac/dkv091

26. Alsan M, Schoemaker L, Eggleston K, et al. Out-of-pocket health expenditures and antimicrobial resistance in low-income and middle-income countries: an economic analysis. *Lancet Infect Dis* 2015;15(10):1203-10. doi: 10.1016/S1473-3099(15)00149-8

27. Selvaraj S, Karan A. Deepening Health insecurity in india: evidence from National sample surveys since 1980s. *Economic & Political Weekly* 2009(40):55-60.

28. Laxminarayan R, Chaudhury RR. Antibiotic Resistance in India: Drivers and Opportunities for Action. *PLoS Med* 2016;13(3):e1001974. doi: 10.1371/journal.pmed.1001974

29. Laxminarayan R, Matsoso P, Pant S, et al. Access to effective antimicrobials: a worldwide challenge. *Lancet* 2016;387(10014):168-75. doi: 10.1016/S0140-6736(15)00474-2

30. Gandra S, Mojica N, Klein EY, et al. Trends in antibiotic resistance among major bacterial pathogens isolated from blood cultures tested at a large private laboratory network in India, 2008–2014. *International Journal of Infectious Diseases*;50:75-82. doi: 10.1016/j.ijid.2016.08.002

31. Datta S, Wattal C, Goel N, et al. A ten year analysis of multi-drug resistant blood stream infections caused by Escherichia coli & Klebsiella pneumoniae in a tertiary care hospital. *Indian J Med Res* 2012;135(6):907-12.

32. Laxminarayan R, Duse A, Wattal C, et al. Antibiotic resistance-the need for global solutions. *Lancet Infect Dis* 2013;13(12):1057-98. doi: 10.1016/S1473-3099(13)70318-9

33. Chandy SJ, Naik GS, Balaji V, et al. High cost burden and health consequences of antibiotic resistance: the price to pay. *J Infect Dev Ctries* 2014;8(9):1096-102. doi: 10.3855/jidc.4745

34. Holstiege J, Schink T, Molokhia M, et al. Systemic antibiotic prescribing to paediatric outpatients in 5 European countries: a population-based cohort study. *BMC Pediatr* 2014;14:174. doi: 10.1186/1471-2431-14-174

35. Suda KJ, Hicks LA, Roberts RM, et al. Trends and seasonal variation in outpatient antibiotic prescription rates in the United States, 2006 to 2010. *Antimicrob Agents Chemother* 2014;58(5):2763-6. doi: 10.1128/AAC.02239-13

36. Kotwani A, Wattal C, Katewa S, et al. Factors influencing primary care physicians to prescribe antibiotics in Delhi India. *Fam Pract* 2010;27(6):684-90. doi: 10.1093/fampra/cmq059

37. Holloway KA, Kotwani A, Batmanabane G, et al. Antibiotic use in South East Asia and policies to promote appropriate use: reports from country situational analyses. *BMJ* 2017;358:j2291. doi: 10.1136/bmj.j2291

38. NCDC. National Treatment Guidelines for Antimicrobial Use in Infectious Diseases. New Delhi: National Center for Diease Control, 2016.

39. Walia K, Ohri VC. Strengthening surveillance key to addressing antimicrobial resistance. *Indian J Med Microbiol* 2016;34(4):413-15. doi: 10.4103/0255-0857.195378

40. CDSCO. Implementation of the Drugs and Cosmentics (Fourth Amendment) Rules. In: Organization CDSC, ed. New Delhi, 2014.
41. MOHFW. Will achieve full immunization coverage for all children by 2020 through a Catch-Up campaign. New Delhi: Press Information Bureau 2014.

Supporting information

S1 Table. Antibiotic prescription rate per 1000 person per year, by age group, India (2013-2014)

S2 Table. Distribution of outpatient antibiotic prescriptions by disease conditions and antibiotic classes, India (2013-2014)

Fig 1

Click here to download Figure Fig 1.tiff

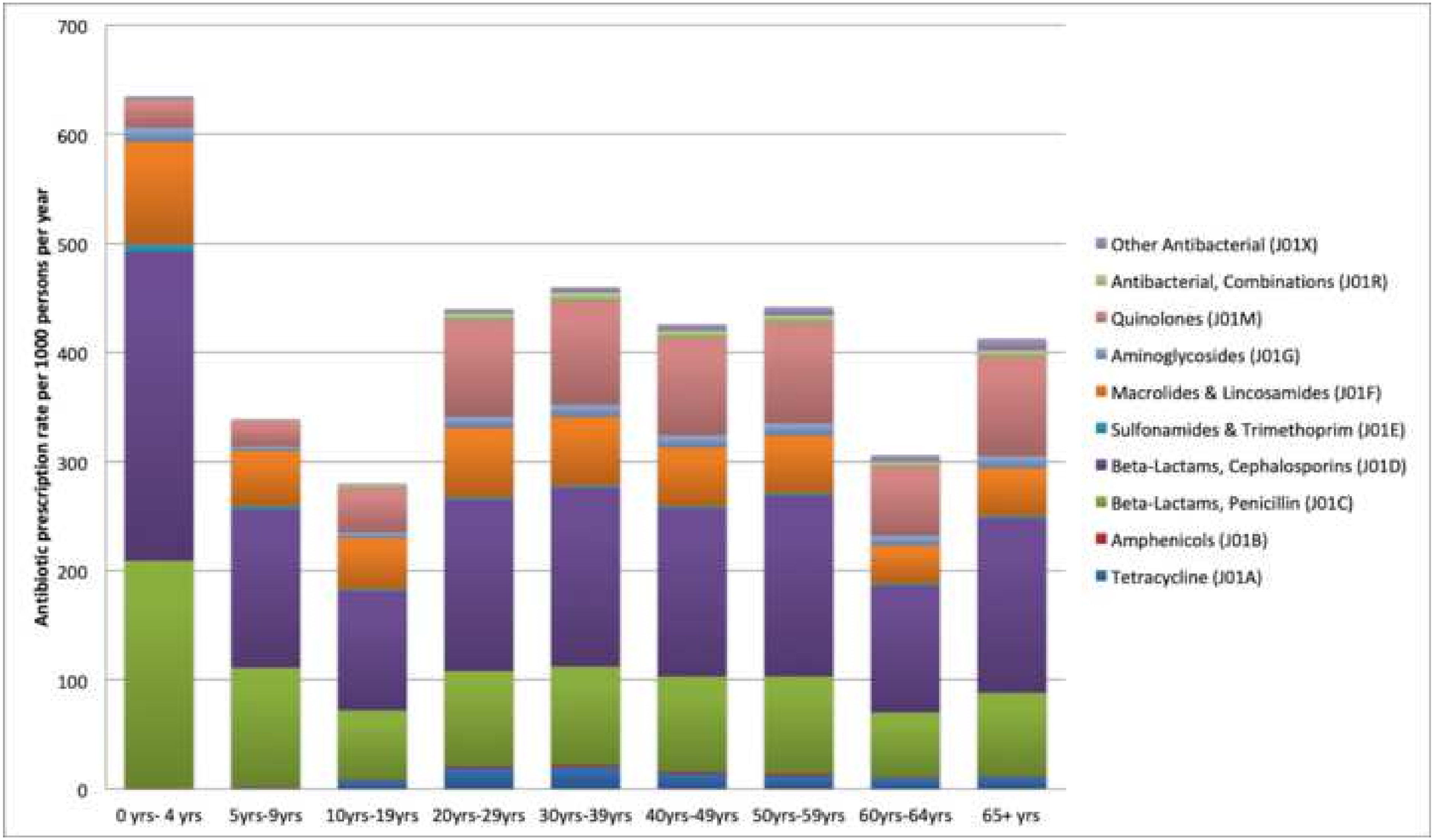

Fig 2

Click here to download Figure Fig 2.tiff

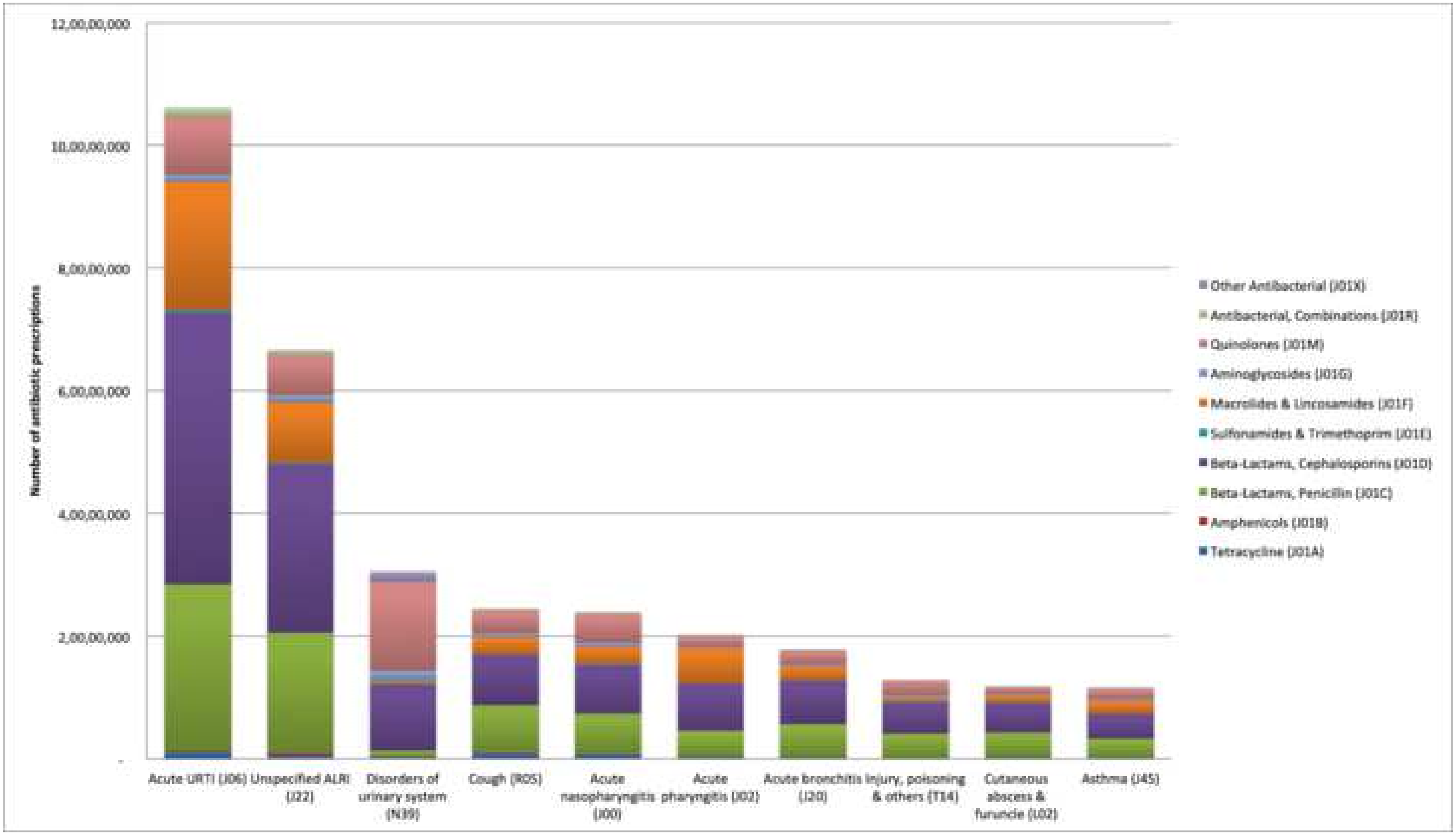

Click here to access/download
Supporting Information
Supporting information.docx
