# Supplementary material for: Outpatient antibiotic prescription rate and pattern in the private sector in India: Evidence from medical audit data
Source: PLoS One. 2019 Nov 13;14(11):e0224848. doi: 10.1371/journal.pone.0224848 (PMC6853304; doi:10.1371/journal.pone.0224848)
Supplement: Attachment — Submitted filename: Outpatient antibiotic prescription rate_PLOSOne_review.doc [file pone.0224848.s004.doc]

**Review PLOS One**

**Outpatient antibiotic prescription rate and**

**pattern in the private sector in India: Evidence**

**from medical audit data**

This study proposed to estimate outpatient antibiotic prescription rate and patterns in the private sector in India during one year using one medical audit dataset from IMS Health (now IQVIA). The relevance of this study is the increasing room taken by the big data in healthcare sciences and the switch of paradigm that will be linked to this evolution.

This manuscript exposed their automated method to estimate antibiotic prescription rates and patterns of prescription according to coding practice in a sample of medical reports from the outpatient private sector in India. To conclude the study reported high antibiotic prescription level overall, by antibiotic classes, age groups, and ICD-10 mapped disease conditions.

The topic is highly relevant because antibiotic consumption remains a major public health problem worldwide, promoting the spread of antimicrobial-resistant organisms. Moreover, to measure and survey the antibiotic prescription rate could represent an added value in combination with infection control strategies, for both achieving successful outcomes in patients and impacting the rate of prescriptions.

Although this approach is meriting, the issues of the paper are not meaningful enough and convincing. I do not agree with the conclusion of the authors: data results could not lead to the conclusion “We observed an inappropriate and high antibiotic prescription rates

for upper respiratory infections in children age less than 5 years”. Indeed, you used a dataset to estimate the rates and the motif of prescription; to prescribe an antibiotic is associated with a lot of arguments and to estimate if appropriate or not you must read the entire medical report, with biological results (you did not include) the symptoms, the X-rays and so on. To use retrospective medical and clinical data to estimate the rate of AB prescription is a tool we have to develop nowadays, but you can’t conclude on pertinence with this kind of methods.

There are different potential methodological reasons for that, the main being that very different clinical presentations could lead to quote a upper respiratory infections; but if the person has comorbidities or other medical condition, maybe an antibiotic could be necessary. You did not adjust your results on the conditions of the patients. The study could be more relevant in presenting the results without this kind of interpretation that can’t be done.

Specific comments

**The introduction** is in my opinion too focused on India, comparisons with outpatient antibiotic prescription in other countries (Western countries but more similar Eastern countries).

**Method section**: it is well written even if some typos remain (cf. manuscript)

Concerning the representativity of your study population, this sample of GP in the private sector in India.

*how are recruited the GPs? Volontary? At random? Request? Please explain

*how could you be sure they are representative of the entire country?

You use the population census as the denominator; however aren’t you afraid of the part of this population going to the public sector of healthcare? And so you used a higher number of persons than the targeted population, giving a misinterpretation of the ab prescription rates? What do you think?

The dataset did not have the motif of the visit and you coded yourself in ICD -10 codes? From wath kind of database? Who did choose the motif of consultation? The pattern of the visit?

This is a little surprising as you went to specific data to structured but less specific ones???

Explain the initial data before the transcription in ICD-10 codes.

How did you take into account the factthat the person could come twice in the same week for the same event and first not have Ab than as still ill, has finally been prescribed one treatment.

**Discussion section**: cf manuscript

A bit too long even if interesting

Some extra subject paragraphs that could be taken out in my opinion or shortened.

**References**:

Some typos and lack of recent literature on that topic. Authors should better screened references databases. Some suggestions to help:

*Antibiotic Prescribing in Outpatient Children: A Cohort From a Clinical Data Warehouse. Grammatico-Guillon L, Shea K, Jafarzadeh SR, Camelo I, Maakaroun-Vermesse Z, Figueira M, Adams WG, Pelton S. Clin Pediatr (Phila). 2019 Jun;58(6):681-690.*

*Durkin MJ, Jafarzadeh SR, Hsueh K, Sallah YH, Munshi KD, Henderson RR, et al. Outpatient Antibiotic Prescription Trends in the United States: A National Cohort Study. Infect Control Hosp Epidemiol. 2018 Feb 27;1–6.*

*Hersh AL, Jackson MA, Hicks LA, the COMMITTEE ON INFECTIOUS DISEASES. Principles of Judicious Antibiotic Prescribing for Upper Respiratory Tract Infections in Pediatrics. PEDIATRICS. 2013 Dec 1;132(6):1146–54.*
